# 3D nano-structures for laser nano-manipulation

**DOI:** 10.3762/bjnano.4.62

**Published:** 2013-09-17

**Authors:** Gediminas Seniutinas, Lorenzo Rosa, Gediminas Gervinskas, Etienne Brasselet, Saulius Juodkazis

**Affiliations:** 1 Centre for Micro-Photonics, Faculty of Engineering and Industrial Sciences, Swinburne University of Technology, Hawthorn, VIC 3122, Australia; 2 The Australian National Fabrication Facility – ANFF, Victoria node, Faculty of Engineering and Industrial Sciences, Swinburne University of Technology, Hawthorn, VIC 3122, Australia; 3 Laboratoire Ondes et Matière d’Aquitaine (UMR5798) Université Bordeaux 1, 351 Cours de la Libération, 33405 Talence, France

**Keywords:** extraordinary transmission, near field, optical tweezing, plasmonics, reactive ion etching, self-induced back-action

## Abstract

The resputtering of gold films from nano-holes defined in a sacrificial PMMA mask, which was made by electron beam lithography, was carried out with a dry plasma etching tool in order to form well-like structures with a high aspect ratio (height/width ≈ 3–4) at the rims of the nano-holes. The extraordinary transmission through the patterns of such nano-wells was investigated experimentally and numerically. By doing numerical simulations of 50-nm and 100-nm diameter polystyrene beads in water and air, we show the potential of such patterns for self-induced back-action (SIBA) trapping. The best trapping conditions were found to be a trapping force of 2 pN/W/μm^2^ (numerical result) exerted on a 50-nm diameter bead in water. The simulations were based on the analytical Lorentz force model.

## Introduction

Optical trapping is a fundamental experimental technique for physics and biology, which allows to precisely control and position micrometer-sized objects such as dielectric parts for nano-assembly, and biomaterials such as cells and bacteria, through the use of gradient forces, which originate from the interaction with a focused laser beam [1].

Nano-focusing and light control, which are possible with metallic plasmonic structures, are very attractive to engineer optical traps, in order to accurately position and manipulate objects down to the nanometer-scale [2]. Plasmonic nano-antennas have been designed for the trapping of dielectric and metallic particles in the size range of 10 nm [3–5], while fishnet metamaterials have been shown to optically pull 100-nm dielectric beads in simulations [6]. Double nano-hole structures have been built to trap yet smaller 12-nm objects [7], and later even objects of single-protein size [8]. When nanometer-sized objects are handled by this technique, the trapping force weakens and the trap loses stability due to Brownian motion. This forces the laser intensity to be increased up to the point where damage occurs to the delicate biomaterials.

Self-induced back-action (SIBA) [9] has recently emerged as a promising technique to address this shortcoming. The main feature of SIBA trapping is the contribution of the object in defining the optical field distribution in the trap as well as the trapping conditions through focusing/diffraction and the interaction with the localized field on the substrate surface. This opens new degrees of freedom to the device, which can be exploited to reduce the optical intensity required for trapping, and thus to prevent damage to the biological material. The trapping position can also be moved farther away from the device, which provides access to the object from all directions. This technique has been employed with photonic crystals to trap spheres in nano-cavities [10] and to control the translation and the rotation of nano-rods by using resonators etched in waveguides [11].

SIBA trapping and slot-guiding of nano-particles using plasmonic devices [12–13] can be realized by using patterns of hole arrays and grooves. The extraordinary transmission in plasmonics can be exploited in metal hole arrays (MHA) at visible and IR spectral wavelengths as a promising method to introduce narrow-band wavelength-selective filtering [14]. A combination of extraordinary transmission and trapping is a promising direction for handling, moving, and sorting nano-materials. By the creation of patterns, which make plasmonic nano-particles chiral, one can envisage the creation of a controlled delivery of force and torque, hence, nano-hands. Recently, it was demonstrated that even optical pulling can be exerted using plasmonic nano-structures [15].

The fabrication of large areas filled with three-dimensional (3D) nano-structures with SIBA functionality requires a multi-step processing and is slow in throughput. Here, we show a parallel processing route for the fabrication of 3D nano-well structures over large areas of cross-sections in the sub-mm range. We explore experimentally and numerically whether extraordinary transmission [16] can be controlled in terms of intensity and spectral width using such 3D nano-well patterns, and we show the suitability of the fabricated structures for SIBA trapping.

## Experimental

**Figure 1:**
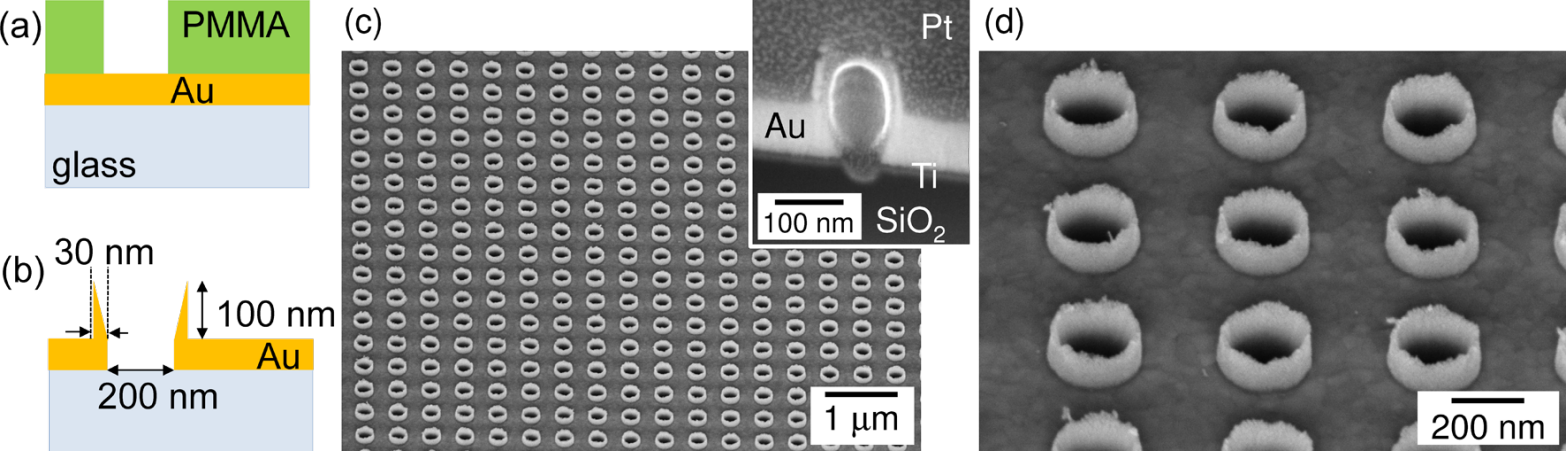
Illustration of the nano-well fabrication by (a) dry plasma etching of Au-film and PMMA mask, which results in Au re-sputtering and (b) formation of a nano-well around the rim of the hole. The experimental outcome is shown as (c) SEM image of the 3D Au nano-wells array; in (d) the magnified slanted view shows details of the nano-well edges. The inset in panel (c) shows the cross-section of a nano-well after deposition of a protective Pt layer.

Gold re-sputtering was carried out for 3.5 min in Ar plasma (Samco RIE-101iPH) at a bias power of 200 W and at a process pressure of 2 Pa. The inductively coupled plasma (ICP) etching mode was turned off. Patterns on a sacrificial 300-nm thick PMMA mask were defined by electron beam lithography (EBL; Raith 150^TWO^). The mask was spin coated on a cover glass which was magnetron sputter-coated (AXXIS, JKLesker) with a 100 nm thick gold film. Figure 1a and Figure 1b show a sketch of the sample structure before and after the re-sputtering step, with conical well structures formed at the rim of holes in the Au film. The plasma etching rate of PMMA was approximately 2.0–2.5 times higher than that of Au. The opening of the holes in the Au-film and the structural quality of samples were characterized by scanning electron microscopy (SEM).

## Numerical simulations

The structure was modeled as a glass substrate on which a gold layer of 100 nm thickness was patterned with a lattice of water-filled (*n* = 1.33) holes of 200 nm diameter. The nano-wells were placed on top of the holes as 100 nm high cylindrical gold rings filled with water. Two cases were considered: one with cylindrical rings of 30 nm wall thickness, and one with conical rings, in which the wall thickness tapers from 30 nm at the bottom to zero at the top, leaving the conical shape on the inside (Figure 1b). The refractive index spectra of the materials were fitted from the experimental values obtained in literature, by means of a built-in polynomial model.

The finite-difference time-domain (FDTD) included a section of the substrate, enclosed in all directions by perfectly matched layers (PML) to avoid spurious reflections. The central area of 5×5 periods was illuminated by a total-field/scattered-field (TFSF) source, in order to measure separately the total field in the central area, and the field scattered outside and to calculate the cross-sections. For the calculation of transmitted and reflected power by the substrate, the domain was changed to a plane-wave illumination from the bottom, also modifying the lateral domain boundaries to periodic boundary conditions (PBC) to avoid diffraction of the plane wave from the borders. In this case, the reflected and transmitted power were measured by two power monitors, placed at either end of the domain, to gather evidence of extraordinary transmission. For the force calculation, the trapped object (a polystyrene bead) was introduced in the total-field region, and surrounded by a 3D monitor recording the vectorial E- and H-fields, discriminating the object volume by the refractive index change, and applying the Lorentz force formulation explained in the following section. The source was linearly *x*-polarized with a bandwidth range from 400 to 1400 nm.

The simulations were performed on the swinSTAR supercomputer at Swinburne University with 16-core nodes of 64 GB memory each. Each simulation took about 1 hour on a 16-node cluster with 256 total cores.

## Background: Lorentz force

The Lorentz force calculation is presented below for the “bound” and “free” charges and currents as *ρ*_b,f_ and **j**_b,f_, respectively. Let us consider a monochromatic field and consider nonmagnetic media only. Using the complex representation for the light field, the real Lorentz force density [N·m^−3^] is:

[1]
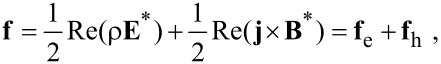


where ρ = ρ_b_ + ρ_f_ and **j** = **j**_b_ + **j**_f_ are the total charge densities.

First, let us consider the case of pure dielectrics without a free charge or a free current density. On the one hand, we have ρ_b_ = −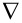 · **P** and ρ_f_ = 0, hence 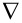 · **D** = 0 with **D** = ε_0_ε_r_**E** = ε_0_**E** + **P** where **P** is the material polarization due to the bound charges and ε_r_ is complex if the medium is not transparent. On the other hand, we have **j**_f_ = 0, **j**_b_ = *∂*_t_ **P**, which leads to:

[2]
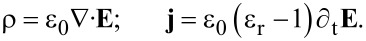


For materials whose dielectric permittivity accounts for conduction electrons, i.e., metals, one would have ρ_b_ = −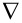 · **P** and 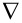 · **D** = ρ_f_. On the other hand, 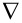 × **H** = **j***_f_* + ε_0_ε*_r_∂_t_***E** and **j***_b_* = ∂*_t_***P** = ε_0_(ε*_r_* − 1)*∂_t_***E**. Therefore, we obtain:

[3]
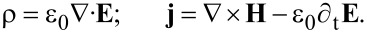


The 3D monitor in the FDTD returns the **E** and **H** fields and we calculate **B** = μ_0_**H**, and, supposing linear media, **D** = ε_0_ε_r_**E**. At the interface between metal and dielectric, the component of **E** parallel to the interface is continuous. The component of **D** perpendicular to the interface must also be continuous, which means the perpendicular component of **E** must be discontinuous and generate a charge density ρ = ρ_b_ + ρ_f_.

When calculating the charge density, ρ, through the divergence of the electric field, one must take into account an unavoidable feature of the FDTD method in that each field component (*E_x_*, *E_y_*, *E_z_*, *H_x_*, *H_y_*, *H_z_*) is calculated for a different point of the Yee cell. As it was recently shown [17], an interpolation scheme can be used to estimate the field at the boundary. Similarly, for the magnetic force, the cross product calculation requires to interpolate the **B** vector components in the positions where the components of **j** are defined. In this case, both *B_x_* and *B_y_* are discontinuous at the interface. However, with the employed scheme [17] the interpolation error only affects the magnetic *z*-component of the Lorentz force 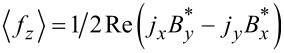.

The total force per unit volume 
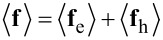
 is then integrated on the trapped object volume 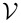 obtaining

[4]
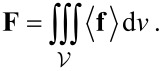


The Maxwell stress tensor (MST) approach can also be used for force calculation, however, there is an ambiguity in the placement of the monitors across the interfaces [17]. In case of the method outlined here, the 3D field monitors, which capture the complex values of the fields provide direct means to remove the ambiguity and calculate the Lorentz force. The analytical formulas presented here were implemented in the simulations.

## Results and Discussion

### Optical transmission

Figure 1c and Figure 1d show SEM images of the fabricated structures. Following the usual PMMA resist development procedure after dry etching, the formation of uniform well-like structures was observed at the rim of the etched holes. The structures were approximately 100 nm in height and about 20–30 nm in width at the base. Their formation can be understood as a re-deposition of the sputtered gold film. During sputtering some gold is deposited on the inner walls of the openings in the sacrificial PMMA layer. This results in a free-standing pattern with enough structural strength to withstand the wet-bath development. A lengthy one-hour treatment at 70 °C in an ultrasonic bath would be necessary to remove these nano-well structures. The wells are made of Au as confirmed by using energy dispersive X-ray spectroscopy using tilted slices, as shown in the inset of Figure 1c.

**Figure 2:**
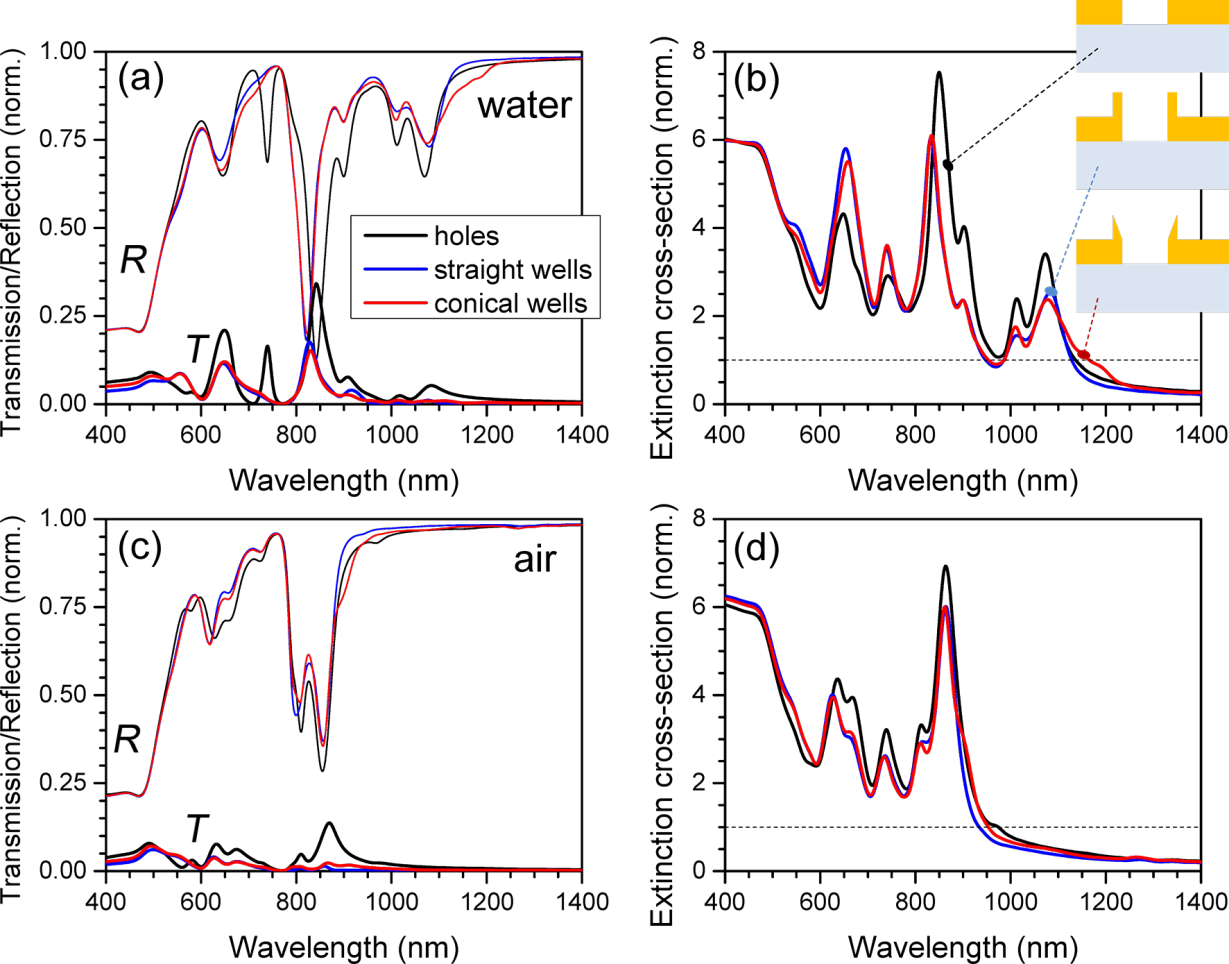
(a) Transmission *T* and reflectivity *R* coefficients for different geometries of the hole/well-array structures (see Figure 1 for geometry: hole diameter *D* = 200 nm, well height *h* = 100 nm, width of the well structure at the base *w* = 30 nm, and thickness of Au film 100 nm). Calculated for immersion in water with refractive index *n*_w_ = 1.33. (b) Extinction cross-sections normalized to the geometrical cross section (dashed line). (c) and (d) are the same as (a) and (b), but for air.

Figure 2a compares transmission and reflectivity spectra of the hole array structure with cylindrical and conical nano-well patterns. The major spectral features are similar, and by tuning the geometrical parameters of the pattern it is possible to maximize the transmission of specific wavelengths. Manifestation of extraordinary transmission can be seen at the major transmission peak around 830 nm. The classical Bethe transmission ratio through a hole of radius *D*/2 in an opaque screen would follow a *T* 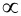 [(*D*/2)/λ]^4^ scaling; for *D* = 200 nm and λ = 800 nm it would be *T* ≈ 0.02 %. By adding the nano-well structure, the spectral properties are not affected strongly and the transmission is reduced. The extinction cross-sections σ_ext_ = σ_abs_ + σ_scat_ defined by the sum of absorption and scattering are shown in Figure 2b. The wavelengths of 760 and 808 nm are chosen for analysis of the light intensity distribution and force mapping (discussed below) because there is no strong pushing (scattering force), which exists at the extraordinary transmission maxima. Also, these are the wavelengths for the planned future laser trapping experiments.

**Figure 3:**
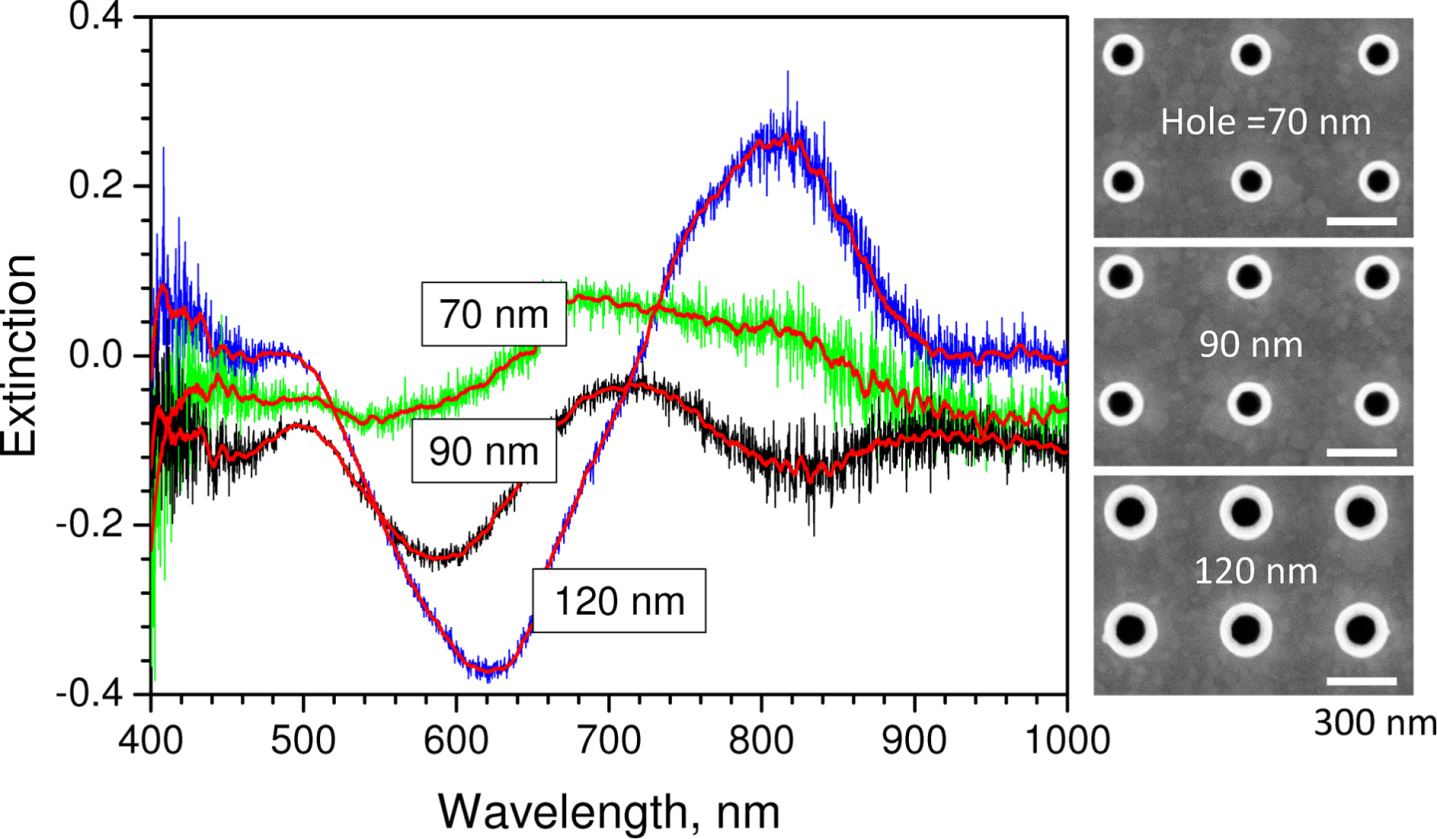
Experimentally measured extinction of the nano-well substrate for hole diameters of 70, 90, and 120 nm. The value is normalized to a continuous 100-nm Au layer, thus the negative regions offer proof of extraordinary transmission [16] through the array.

The transmission of the nano-well arrays was experimentally characterized for hole diameters of 70, 90, and 120 nm, as shown in Figure 3. The extinction, *Ext*, was calculated as *Ext* = ln(*T*_ref_/*T*_sample_), where *T*_ref_ is the transmissivity of a 100-nm Au layer. When the diameter is increased, the extinction peak is red-shifted and becomes negative, which corresponds to extraordinary transmission through the array, which in case of the 120-nm array extends over a wide band between 500 and 730 nm. The spectral tunability of the extinction in the plasmonic band around 600 nm is clearly discernable, and makes it possible to tune the peak up to the laser wavelength to optimize the trapping effect.

**Figure 4:**
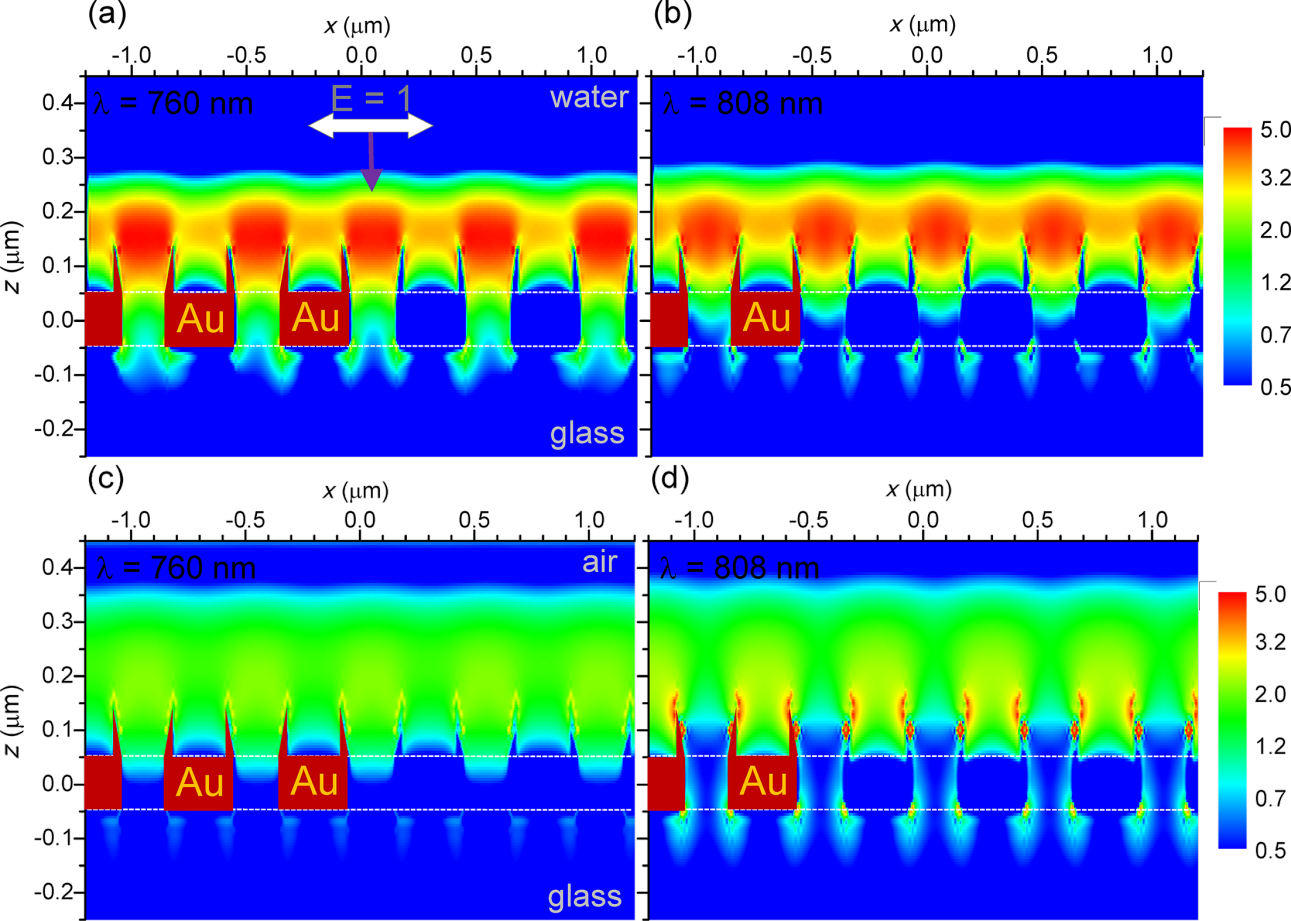
Light intensity distribution in the *xz*-plane simulated by 3D-FDTD at the two laser wavelengths (a,c) 760 nm and (b,d) 808 nm, in (a,b) water and (c,d) air. Incident light intensity |**E**_0_|^2^ = 1; propagation in negative *z*-direction.

The light intensity distributions in the top-illuminated substrates reveals the configuration of the trapping field: while the field enhancement is not very strong (less than 10) in water, as shown in Figure 4a and Figure 4b, we see the formation of large spots hovering above the nano-well apertures: These spots vary little with the wavelength and are useful for trapping dielectric objects. In air the enhancement is much weaker, as shown in Figure 4c, and at the longer wavelength in Figure 4d there occurs the formation of hot-spots at the upper and lower corner of the nano-wells.

Next, we employed the full-3D vectorial model to numerically determine the optical trapping force **F**(**r**,ω) = 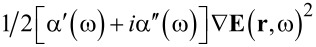 experienced by the bead, which is usually approximated in theory as an optical dipole oscillator, in the vicinity of a nano-well, where (α′ + *i*α″) is the polarizability at the angular frequency ω (linked to the permittivities through the Clausius–Mossotti equation [18]), **r** is the position vector, and 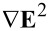 is the E-field intensity gradient [19]. For stable trapping, the force given by the light intensity gradient (proportional to α′) must exceed the force resulting from the momentum transfer by the photons, which are back-scattered by the particle (proportional to α″), and generate a potential minimum low enough to curb the combined effect of gravity, buoyancy, and the Brownian motion of the particle [20]. The polarizability of the nano-materials determines the sign of the force: metallic-like particles are repelled from high intensity regions, while dielectric nano-particles will be attracted.

### Force mapping

**Figure 5:**
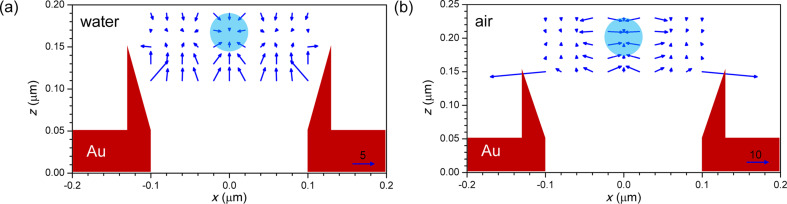
Arrow plot of the trapping force for plane-wave illumination in medium-to-substrate direction (as in Figure 4) at 760 nm wavelength for a 50-nm diameter polystyrene bead in (a) water and (b) air. The conical Au-well profile is schematically shown by the triangular shapes and the circle indicates the position of the bead in the central trap. The scale arrows at the lower right indicate forces of 5 and 10 pN/W/μm^2^.

**Figure 6:**
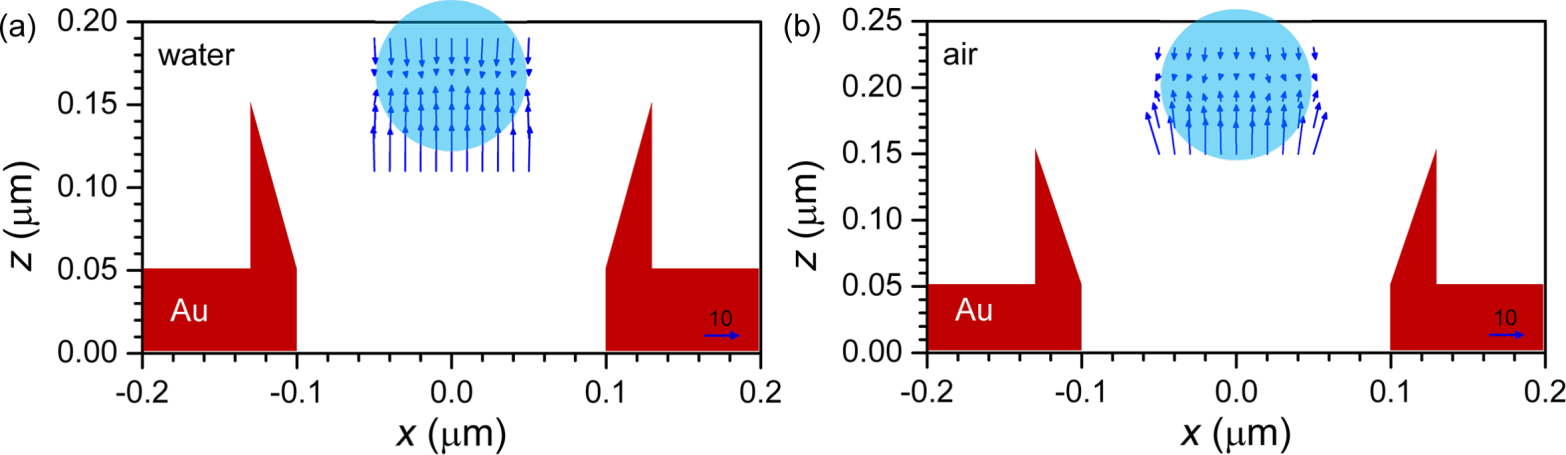
Arrow plot of the trapping force as in Figure 5 at 760 nm wavelength for a 100-nm diameter bead in (a) water and (b) air. The scale arrows at the lower right indicate a force of 10 pN/W/μm^2^.

The force mapping was calculated by using the Lorentz force formalism (from section “Background: Lorentz force”) on a polystyrene-bead probe (*n* = 1.504) of diameter *d* with the 3D-FDTD method. In order to build the map, the bead is moved in 10-nm steps along the *xz*-plane at different heights in the proximity of the top of the well. At each position the simulation is repeated and the 3D Lorentz force is calculated. Due to the density of polystyrene being approximately equal to that of water, buoyancy and gravity almost compensate each other, leaving a residual force about three orders of magnitude lower than the Lorentz force under normal illumination conditions. Figure 5 and Figure 6 show the force field map at the rim of the nano-well.

The best trapping conditions were found for the conical nano-wells at 760 nm wavelength. The trapping locations depicted by arrow plots in Figure 5 for a bead with *d* = 50 nm and Figure 6 for a bead with *d* = 100 nm, show very different equilibria between the transverse force *f_x_* and the longitudinal force *f_z_*. In all cases, there is a central trapping spot at (0,*z*_0_), while the 50-nm bead also shows lateral ones (making a ring at about 50 nm distance from the center) with weaker transverse confinement and a tendency to easily escape the nano-well. This provides a strategy for moving the particles from one well to the neighboring ones by ‘storing’ them in the ring. The trapping height *z*_0_ of about 165 nm in water and about 200 nm in air, measured from the center of the metal layer, is relatively independent of the particle size. However, due to the particle altering the field distribution, the force balance is strongly affected.

The most balanced trapping is achieved in water for the 50-nm particle in Figure 5a, with a maximum force of 2 pN/W/μm^2^ in all directions in the particle vicinity. In air (Figure 5b) the longitudinal confinement is similar, but the lateral confinement is much stronger at 5 pN/W/μm^2^, which makes it more difficult to shift the particle between neighboring wells. In the transverse direction, the smaller particle shows the escape distance from the central trap to be around half of the diameter, with the maximum force occurring at the maximum field gradient, which is consistent with the behavior of a single-beam optical tweezer [21]. One would then expect this distance to be independent of the diameter, as the particle size is much smaller than the wavelength. However for the 100-nm particle in Figure 6 we have a very strong longitudinal force *f_z_* of more than 20 pN/W/μm^2^, but a very weak lateral confinement as the dielectric particle is attracted by the hot-spots along the nano-well walls. Because of this, the particle will tend to move randomly across the well, eventually escaping it in the case of air, while for water-trapping the lower position permits confinement by the walls, as long as the mechanical strength of the metal cone is enough to resist the impact of the particle. The increase in *z*-force with the diameter is also consistent with single-beam tweezers, where the maximal force scales with the bead volume for particles, which are small with respect to the wavelength [20].

**Figure 7:**
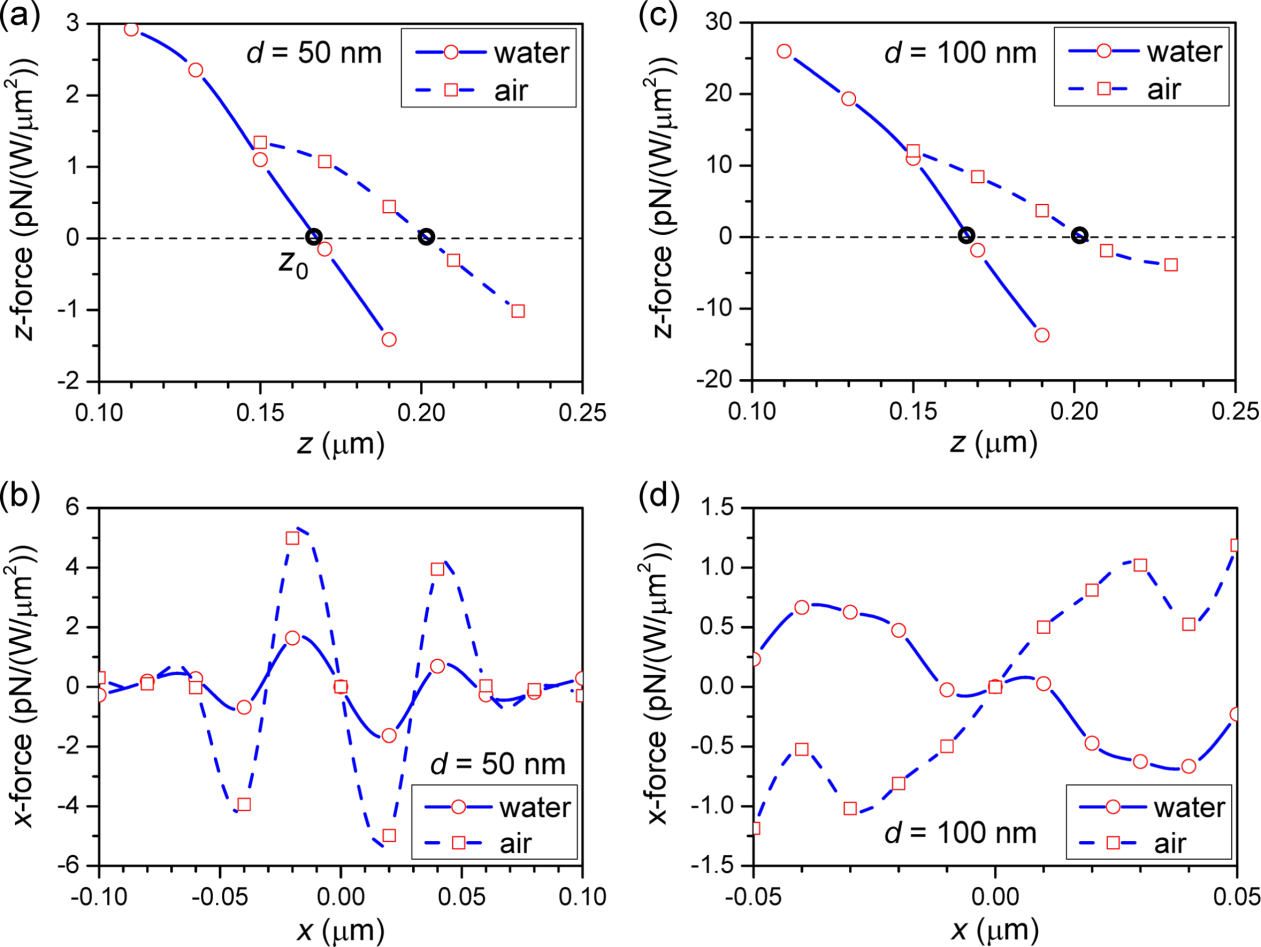
Force evaluation in the neighborhood of the central trapping spot (0,*z*_0_) at 760 nm wavelength for a bead of diameter *d* in water (solid) and air (dashed): (a,c) *f_z_*(*z*) at *x* = 0 and (b,d) *f_x_*(*x*) at *z* = *z*_0_. The calculated values are indicated by the red markers, the blue lines are guides to the eye.

This is confirmed by examining the force components in the trap to calculate the stiffness around the trapping spot [22] as shown in Figure 7. Considering an incident intensity of 1 W/μm^2^, for the longitudinal *z*-direction we see that for the 50-nm bead in Figure 7a, the trap has a stiffness of about 0.06 pN/nm in water and 0.04 pN/nm in air. In the transverse *x*-direction in Figure 7b, the stiffness is about 0.05 pN/nm in water and 0.25 pN/nm in air for the central spot. Because the diameter is small with respect to the wavelength, the stiffness is expected to increase with the diameter, as the amount of material intercepted by the field is larger, which increases the trapping power [21]. However, due to the close SIBA interaction and redistribution of field, the *z*-direction stiffness increases by about one order of magnitude for the 100-nm bead in Figure 7c, while the transverse confinement is very different in Figure 7d at about 0.01 pN/nm in water. In air we notice that the particle is actually pushed away from the center and tends to escape the well, except for a weak confinement ring at a distance of 40 nm from the center.

Because the trapping position is relatively far away from strong field gradients, the escape force in the nano-wells is about one hundredth, and the stiffness is about one tenth, in comparison to the original SIBA trapping-substrate [9]. Yet, they are still significantly stronger than the maximum force given by Brownian motion. However, there is a significant benefit in that the nano-wells trap the particle at a much more exposed position, which makes the particle accessible from all sides to inspection and imaging. In addition the distance from the substrate makes it easier to probe the particle without interfering with the trap.

## Conclusions

We demonstrate a simple plasma etching procedure which produces conical nano-well structures. The potential use in laser-trapping through the SIBA mechanism is numerically corroborated and analytical formulas are presented. The complex extinction spectra of the structures show that wavelength tunable lasers would be the best candidates to explore the peculiarities of nano-tweezing. We can foresee an enhancement of nano-material delivery into regions of high light intensity via Marangoni flow induced by localized heating and convection [23].
